# Multimodal brain age prediction fusing morphometric and imaging data and association with cardiovascular risk factors

**DOI:** 10.3389/fneur.2022.979774

**Published:** 2022-12-14

**Authors:** Pauline Mouches, Matthias Wilms, Agampreet Aulakh, Sönke Langner, Nils D. Forkert

**Affiliations:** ^1^Biomedical Engineering Program, University of Calgary, Calgary, AB, Canada; ^2^Hotchkiss Brain Institute, University of Calgary, Calgary, AB, Canada; ^3^Department of Radiology, University of Calgary, Calgary, AB, Canada; ^4^Department of Paediatrics, Cumming School of Medicine, University of Calgary, Calgary, AB, Canada; ^5^Department of Community Health Sciences, Cumming School of Medicine, University of Calgary, Calgary, AB, Canada; ^6^Alberta Children's Hospital Research Institute, University of Calgary, Calgary, AB, Canada; ^7^Schulich School of Engineering, University of Calgary, Calgary, AB, Canada; ^8^Institute for Diagnostic Radiology and Neuroradiology, Rostock University Medical Center, Rostock, Germany

**Keywords:** cardiovascular risk factors, brain aging, brain age gap, magnetic resonance angiography, magnetic resonance imaging (MRI)

## Abstract

**Introduction:**

The difference between the chronological and biological brain age, called the brain age gap (BAG), has been identified as a promising biomarker to detect deviation from normal brain aging and to indicate the presence of neurodegenerative diseases. Moreover, the BAG has been shown to encode biological information about general health, which can be measured through cardiovascular risk factors. Current approaches for biological brain age estimation, and therefore BAG estimation, either depend on hand-crafted, morphological measurements extracted from brain magnetic resonance imaging (MRI) or on direct analysis of brain MRI images. The former can be processed with traditional machine learning models while the latter is commonly processed with convolutional neural networks (CNNs). Using a multimodal setting, this study aims to compare both approaches in terms of biological brain age prediction accuracy and biological information captured in the BAG.

**Methods:**

T1-weighted MRI, containing brain tissue information, and magnetic resonance angiography (MRA), providing information about brain arteries, from 1,658 predominantly healthy adults were used. The volumes, surface areas, and cortical thickness of brain structures were extracted from the T1-weighted MRI data, while artery density and thickness within the major blood flow territories and thickness of the major arteries were extracted from MRA data. Independent multilayer perceptron and CNN models were trained to estimate the brain age from the hand-crafted features and image data, respectively. Next, both approaches were fused to assess the benefits of combining image data and hand-crafted features for brain age prediction.

**Results:**

The combined model achieved a mean absolute error of 4 years between the chronological and predicted biological brain age. Among the independent models, the lowest mean absolute error was observed for the CNN using T1-weighted MRI data (4.2 years). When evaluating the BAGs obtained using the different approaches and imaging modalities, diverging associations between cardiovascular risk factors were found. For example, BAGs obtained from the CNN models showed an association with systolic blood pressure, while BAGs obtained from hand-crafted measurements showed greater associations with obesity markers.

**Discussion:**

In conclusion, the use of more diverse sources of data can improve brain age estimation modeling and capture more diverse biological deviations from normal aging.

## Introduction

Brain structures are known to undergo morphological changes with normal aging (1, 2), a process that is primarily associated with brain tissue atrophy resulting from a deterioration of neurons and synapses (3). Aside from these macroscopic changes to brain morphology, cerebral blood flow and artery morphology, among others, have also been shown to be affected by aging (4–6). Brain tissue health may be directly associated with the blood flow supplied through main arteries and the downstream exchange of molecules through smaller capillaries. Thus, atrophy and vascular impairments are often seen in tandem with many neurological diseases, such as dementia (7).

Biological brain age estimation using machine-learning models is an important step toward detecting deviation from normal brain aging trends. More precisely, the brain age gap (BAG), representing the difference between the estimated biological brain age and the chronological age of a patient, has been identified as a potential indicator for several neurological diseases (8–10). Thus, it may be a promising biomarker for precision medicine applications in the diagnosis and management of neurological diseases (11). Machine learning models estimating the biological brain age of an individual using hand-crafted, image-derived, morphological features, or brain images directly (9, 12, 13) have been proposed in the past. For morphological hand-crafted features, well-known algorithms such as support vector regression, neural networks, tree-based models, and ensemble-based models have been used in the past (14–16). For brain age estimation using medical images directly, convolutional neural networks (CNN) are typically used. Past studies have proposed different types of CNN architectures with different levels of depth/number of layers and parameters (12, 13), types of convolutional layers [e.g., two-dimensional (9) or three-dimensional (12, 13)], or output types [e.g., age prediction only or bidirectional generative-discriminative models (17, 18)]. However, the type of input data (morphological hand-crafted features or brain images) used by these models has a significant impact on the accuracy of the biological brain age estimation, regardless of the machine learning algorithm used. Some studies, for instance, used the exact same cohort of participants to train two different machine learning models: one using brain magnetic resonance imaging (MRI) scans (images) and one using morphological hand-crafted features extracted from these brain MRI scans as input (19, 20). They observed better accuracy when the brain MRI scans were used directly. Overall, it appears that brain age estimation methods that directly use images generally report better results than those using extracted features (12, 13, 15), though comparing results from studies using different cohorts of participants is not straight forward (21). Practically, models using the image data directly have access to spatial information and internally extract a finite number of high-level features, which are directly optimized toward the prediction task in an end-to-end manner (22). CNNs automatically compute feature maps by convolving the input image with different convolutional kernels, where parameters are optimized during model training. Therefore, CNNs can extract shape or texture features that significantly differ from typically used morphological hand-crafted features (e.g., brain structure volume or thickness).

T1-weighted MRI is widely used for biological brain age estimation as this neuroimaging sequence displays the brain tissues with high contrast. Most previous brain age prediction studies used T1-weighted MRI to compare different predictive models (13, 15, 16). Others found that combining different types of input data derived from T1-weighted MRI could lead to improved brain age prediction results (19, 20, 23). However, using other modalities in addition to T1-weighted MRI may provide additional predictive information for the brain age estimation task. Thus, a few recent studies have combined multiple imaging modalities as input of such models, including, for instance, T2-weighted or diffusion-weighted MRI. These multimodal models generally resulted in overall improved prediction accuracy (24–27). Nevertheless, no comparison between multimodal brain age prediction models using either images or their extracted morphological features as input has been performed so far, although these two types of data might provide substantially different or complementary information. In this specific study, T1-weighted MRI and time-of-flight magnetic resonance angiography (TOF MRA), which contains brain artery information (28), are used. While T1-weighted MRI data have been the de-facto standard modality for brain age prediction, the use of TOF MRA data has only been proposed recently (29, 30), where the TOF MRA imaging data were used as input to CNN models. Furthermore, hand-crafted parameters describing the local artery morphology may add another perspective of valuable information for brain age prediction models. As vascular structures are small and highly variable with respect to their location, CNN models may not be able to fully focus on these structures. Thus, adding this relevant vascular impairment information, which may contribute to normal and pathological aging (7, 31), might improve biological brain age prediction.

Brain age prediction models are usually trained and tested using brain scans from healthy individuals. Models described in the literature typically result in average brain age estimation errors of 3–5 years (8). This clearly illustrates that there is inter-subject variability, even in healthy subjects, that affects biological brain aging. There are many potential reasons for these differences, such as genetic predisposition, environmental factors, the daily diet, drug use including nicotine and alcohol, physical activity, and others. Accounting for all of these factors is likely not possible, especially given that many of these factors can easily change during the course of one's life. However, it may be argued that cardiovascular health is directly affected by many of these parameters, which could be ultimately used as surrogate variables. Within this context, it is well-known that cardiovascular parameters are tightly linked with cognitive decline and dementia (32). Moreover, cardiovascular risk factors have been previously shown to be correlated with the BAG when using biological brain age estimated from morphological hand-crafted features (24, 25, 33, 34), or from images (30, 35). These studies observed significant correlations of BAGs computed using different imaging modalities with several factors such as blood pressure, body-mass index, and smoking. In summary, different imaging modalities seem to capture different biological aging information (33). However, the impact of the input data type (morphological hand-crafted features or images) used to compute the BAG, for a specific imaging modality, on its associations with cardiovascular risk factors has not been investigated yet. Understanding this impact is crucial to evaluate whether morphological hand-crafted features or images, from a given image modality, capture similar or complementary biological brain age information.

Therefore, the aim of this study was to investigate differences between using hand-crafted features and imaging data as input to brain age prediction models, in a multimodal context. To do so, T1-weighted MRI and TOF MRA datasets from 1,658 predominantly healthy adults were used. Morphological hand-crafted features were extracted and used as input to multilayer perceptron (MLP) models while preprocessed imaging data were used as input to CNN models. First, the brain age prediction results of each model were investigated and compared. Then, the benefits of combining brain age predictions from models using morphological hand-crafted features and models using image data directly were analyzed. Finally, associations between the BAGs from the different models and several cardiovascular risk factors were investigated. This study expands on one of our previous studies (29). Briefly described, the aim of this previous study was to use T1-weighted MRI and TOF MRA imaging data to predict brain age and to identify the most predictive regions in the image space. This current study differs from the previous one by performing an in-depth analysis of the value of adding hand-crafted features for the brain age prediction task instead of using imaging data only. Moreover, a detailed analysis of the relationship between the BAG and cardiovascular risk factors is conducted in this study, showing relevant differences between models. Thus, the major contributions of this study are: (i) a thorough comparison of brain age prediction models using morphological hand-crafted features and imaging data, with a focus on multimodal data including TOF MRA datasets; and (ii) the investigation of the relationship between cardiovascular risk factors and BAG computed using different input data types (morphological hand-crafted features or images) and imaging modalities (T1-weighted MRI vs. TOF MRA).

## Materials and methods

### Clinical and imaging data

**Figure 1:**
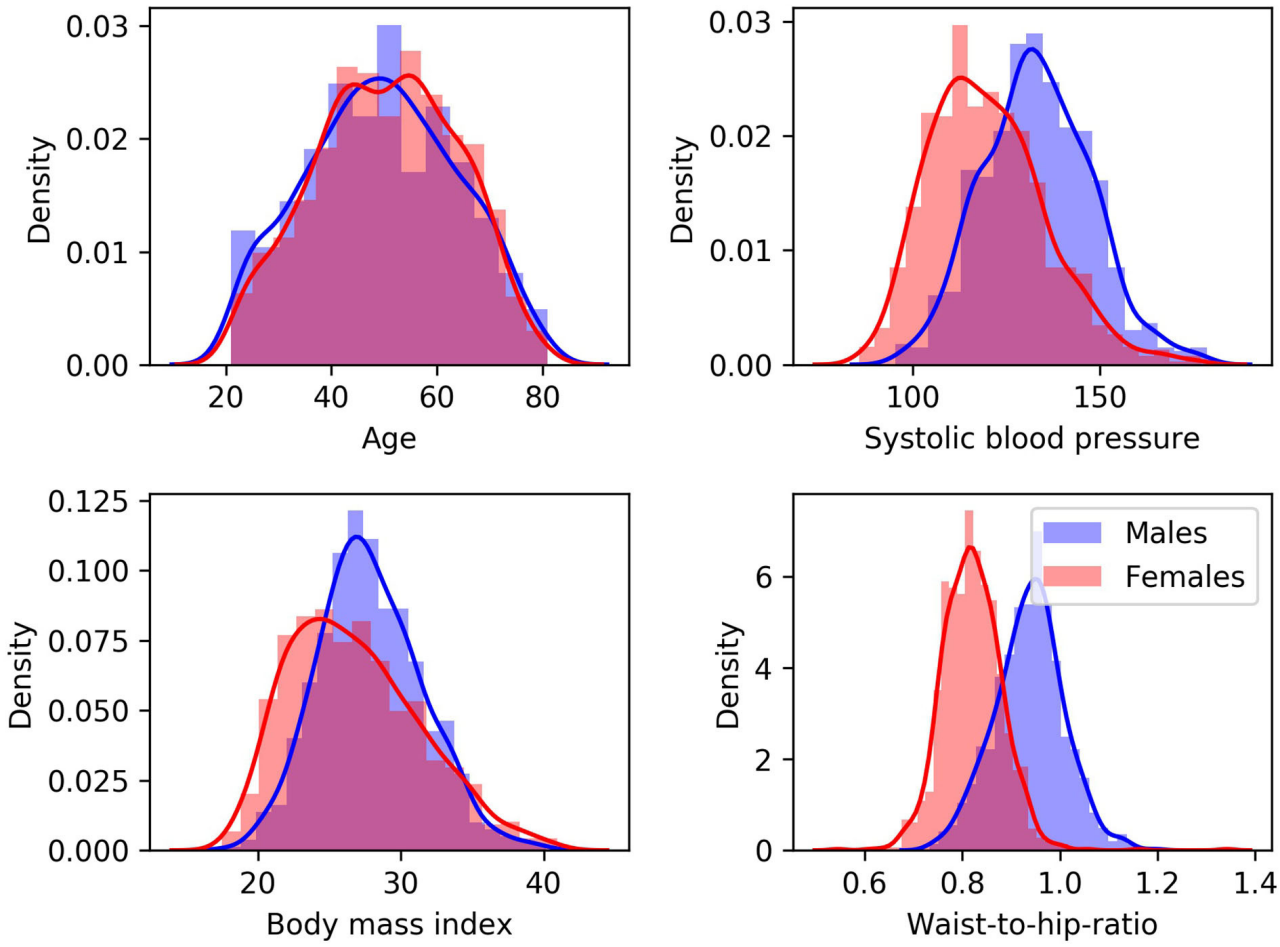
Density plots for males (blue) and females (red) for cardiovascular risk factors with continuous values.

Data from the Study of Health in Pomerania (SHIP), containing randomly selected participants from the region of Pomerania in Germany, were used for this secondary work. The SHIP study aimed to collect data representing the general population to assess the incidence and prevalence of common risk factors as well as subclinical and clinical diseases (36). The data sample used in this second study includes cross-sectional data. After quality control of the imaging data and all pre-processing steps described below, data from 1,658 predominantly healthy adults, aged between 21 and 81 years, were included. T1-weighted MRI and TOF MRA datasets were acquired for each participant using a single 1.5T MRI scanner (Magnetom Avanto; Siemens Medical Solutions, Erlangen, Germany). The acquisition parameters were: T1-weighted MRI: TR = 1,900 ms, TE = 3.4 ms, flip angle = 15°, spacing = 1.0 × 1.0 × 1.0 mm^3^; TOF MRA: TR = 23 ms, TE = 7 ms, flip angle = 25°, spacing = 0.7 × 0.7 × 0.7 mm^3^. Additionally, several clinical, lifestyle, and behavior variables, which are referred to as cardiovascular risk factors in the following, were assessed for each participant. The variables used for this study include body-mass index (BMI) (kg/m^2^), waist-to-hip ratio (WHR), systolic blood pressure (BP) (mmHg) averaged over three measurements, smoking history (encoding the following information: smoker vs. non-smoker; past vs. current smoker; regular vs. occasional smoker), and alcohol consumption (number of glasses of alcohol per week). The cardiovascular risk factors are summarized in Table 1 and Figure 1, which shows density plots for the cardiovascular risk factors with continuous values.

**Table 1:** Demographic and cardiovascular risk factor information of the participants included in the study.

	**Mean (std)**
**Clinical factors:**	
Age (years)	49.9 (13.7)
Sex (**F:** female; **M:** male)	**F:** 909; **M:** 749
Systolic blood pressure at rest (mmHg)	126.0 (16.4)
Body-mass index (BMI; kg/m^2^)	27.3 (4.2)
Waist-hip ratio (WHR)	**F:** 0.82 (0.06); **M:** 0.94 (0.07)
**Behavioral factors:**	
Smoking status (0: never smoked; 1: past occasional smoker; 2: past regular smoker; 3: current occasional smoker; 4: current regular smoker)	**0:** 668; **1:** 56; **2:** 347; **3:** 266; **4:**321
Number of glasses per week (**0:** 0; **1:** < 1; **2:** 1–6; **3:** **>**6)	**0:** 112; **1:** 458; **2:** 963; **3:** 125

All participants provided written informed consent and the SHIP study was approved by the local ethics commission of the University of Greifswald (BB 39/08, 19.06.2008). The scans were completely anonymized for this secondary study so that no additional ethics approval was required.

### Morphological hand-crafted features: Extraction and model architecture

**Figure 2:**
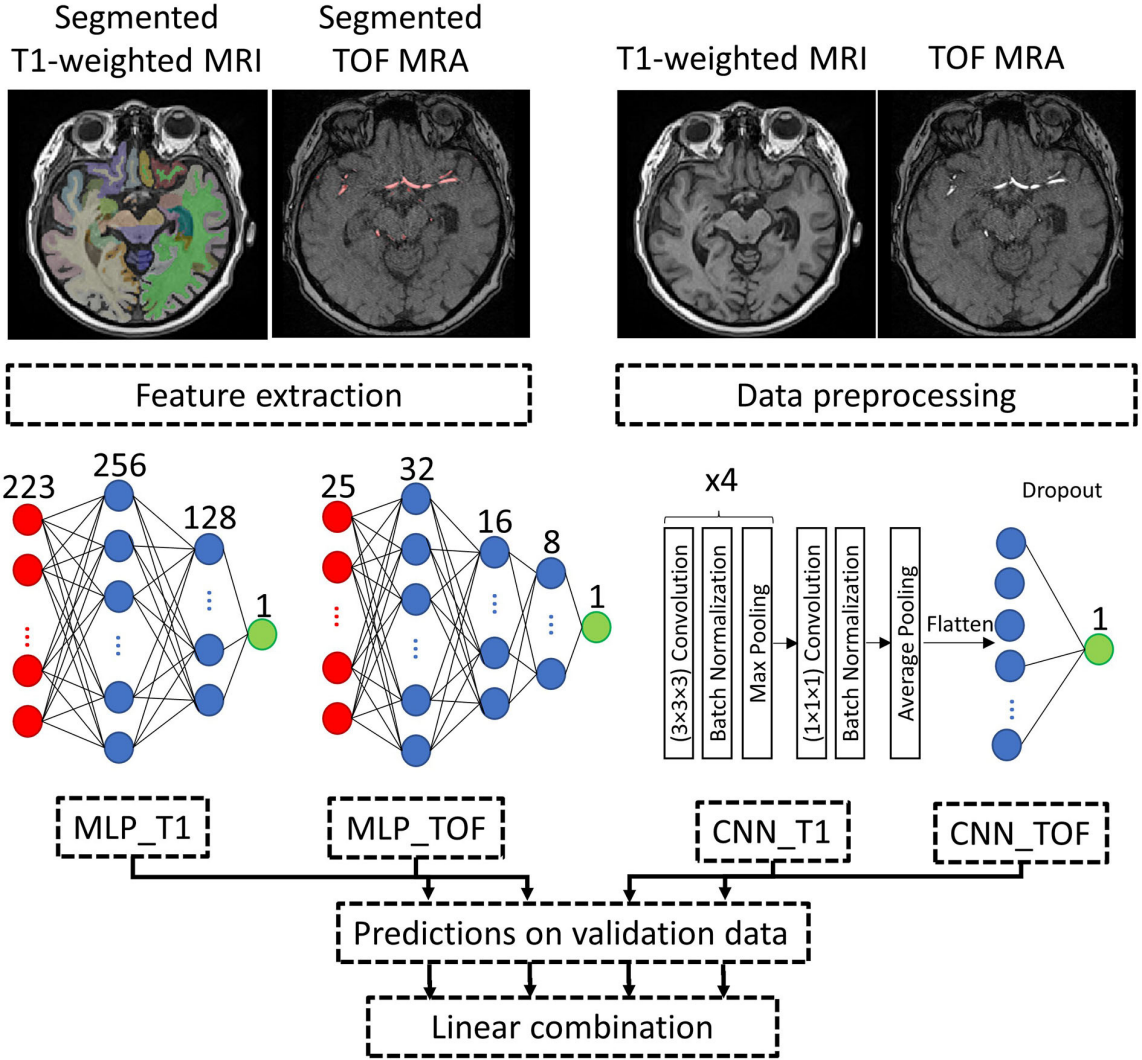
Processing pipeline. Example input data and approach to combine the brain age prediction models using different input types. MLP and CNN model architectures are detailed, with both CNNs (CNN_T1 and CNN_TOF) having the same model architecture. Red: input layer, Blue: hidden layer, Green: output layer. MLP, Multilayer perceptron; CNN, Convolutional neural network; T1-weighted MRI, T1-weighted magnetic resonance imaging; TOF MRA, Time-of-flight magnetic resonance angiography.

Figure 2 illustrates an overview of the input data processing, model architecture, and model combination approach.

Morphological brain features were extracted from the T1-weighted MRI brain scans using FastSurfer (37). FastSufer is a deep learning pipeline replicating the widely used Freesurfer (38) analysis pipeline with reduced computational requirements. It segments a T1-weighted MRI dataset into 95 brain structures, and the extracted features include the surface area, gray matter volume, and average thickness of cortical structures, as well as the volume of subcortical structures and the total brain volume. As a result, 223 features were extracted and are available for each participant.

Morphological artery characteristics were extracted from the TOF MRA datasets. Therefore, a level-set-based method was first used to segment the arterial structures from the TOF MRA datasets. Briefly described, this method uses a fuzzy combination of the TOF MRA intensity image and the vessel-enhanced TOF MRA image (39) as input to a level-set segmentation algorithm with anisotropic energy weights (28). Then, the centerline of each vessel was extracted using the method described in Lee et al. (40) and further used to estimate the vessel thickness. Vessel thickness was calculated by determining the distance between each centerline voxel and the closest vessel boundary voxel, estimated using the distance transform described in Danielsson (41). Two types of regions of interest, defined in the MNI brain atlas space (42), were used: the cerebral blood flow territories (43), and the major brain arteries (6), which were localized using a statistical TOF MRA atlas (44). These regions of interest were transformed into each individual TOF MRA dataset by combining image registration transformations, as detailed in Mouches et al. (6). Generally, two transformations were estimated: a rigid transformation between each TOF MRA dataset and its corresponding T1-weighted MRI dataset, and a non-linear transformation between each T1-weighted MRI dataset and the 152 MNI brain atlas. The transformed atlas regions were used together with each corresponding vessel segmentation and vessel thickness map to extract region-specific artery measurements. The final set of features includes the diameters of the main arteries, including the posterior cerebral arteries (PCA), the middle cerebral arteries M1 and M2 segments (MCA), the basilar artery (BA), the internal carotid arteries (ICA), and the anterior cerebral arteries A1 and A2 segments (ACA). Furthermore, the mean artery diameter and density in the major blood flow territories (MCA, PCA, and ACA territories) were quantified, resulting in a total of 24 features.

The quality of the datasets and the corresponding segmentations were visually checked by an observer with more than 5 years of dedicated experience in brain image analysis (PM). For T1-weighted MRI datasets, FastSurfer segmentation results were overlaid onto each MRI scan and evaluated in axial, coronal, and sagittal views. Datasets with insufficient segmentation results for at least one brain structure were excluded. Linear registration of the T1-weighted MRI was assessed by overlaying each dataset onto the MNI brain atlas and excluding mis-registered datasets. For the TOF MRA datasets, quality control was conducted in two steps. First, segmentation accuracy was visually assessed using a three-dimensional visualization of the segmented vessels. Then, the registration of the MNI brain atlas to each TOF MRA dataset was visually checked by overlaying the registered cerebral blood flow territories and major brain artery masks onto the vessel segmentations for each participant. Exclusion criteria were incomplete or noisy vessel segmentation and mis-registration, as described in Mouches et al. (6). Figure 2 shows the segmentation results for one dataset.

Two separate MLP models were used to predict brain age from the morphological hand-crafted features extracted from the T1-weighted MRI (223 features) and the TOF MRA (24 artery features + total brain volume) datasets. The architecture of the MLP models was optimized by testing different configurations with one to four hidden layers and the following number of neurons per hidden layer: T1-weighted MRI: (256), (256, 128), (256, 128, 64), (256, 128, 64, 32); TOF MRA: (32), (16, 32), (8, 16, 32), (4, 8, 16, 32). The MLP models were trained for 1,000 epochs using the Adam optimizer with a learning rate of 0.1 and a batch size of 200.

### Image data: Preprocessing and model architecture

The T1-weighted MRI datasets as well as the TOF MRA datasets were pre-processed to facilitate brain age prediction using the images directly as input. More precisely, the pre-processing steps included bias field correction (45), skull stripping (46), and affine registration to the MNI brain atlas (42), using the registration algorithm implemented in ANTs (47). Finally, the intensity of each scan was center scaled using its mean intensity and standard deviation and the scans were cropped to remove non-informative background voxels. More information about the preprocessing steps can be found in (29).

The architecture for the CNN models used in this work was inspired by the Simple Fully Convolutional Neural Network (SFCN) proposed in Peng et al. (13), which is one of the best performing model architectures when trained and evaluated using the UK Biobank data (48). Briefly described, the model architecture contains four consecutive blocks consisting of one three-dimensional convolutional layer [(3 × 3 × 3) kernel], one batch normalization layer, one max pooling layer [(2 × 2 × 2) kernel], and one ReLU non-linear activation layer (49). The convolutional layers from these four blocks contain 32, 64, 128, and 256 filters, respectively. A fifth block contains one three-dimensional convolutional layer with a (1 × 1 × 1) kernel and 64 filters, and one batch normalization layer followed by ReLU activation. Finally, a sixth block consisting of an average pooling layer, a dropout layer with a 0.5 dropout rate, and a dense layer with linear activation outputs the age prediction. The models were trained from scratch to predict the brain age using a mean squared error as a loss function and the Adam optimizer with a learning rate of 0.001, a batch size of 8, and 200 epochs. Additionally, data augmentation consisting of ± 5° rotations and ± 10 voxel translations was applied to 50% of the training data in each batch to prevent overfitting and increase model performance (29).

### Model evaluation

The models were evaluated using an age-stratified 5-fold cross-validation approach. Therefore, for each iteration of the cross-validation, 1-fold was used for testing and 4-folds were used for training. Two hundred forty-nine datasets from each training set (15% of the whole data) were selected in an age-stratified way for validation. This approach ensures the robustness of the results by leveraging a large amount of training data in each cross-validation iteration while using each dataset for testing once. The best models for each fold were obtained by monitoring the loss value on the validation data during training and saving the model with the lowest loss value. The best performing architecture for the MLP models, on average over the 5-folds, had 2 and 3 hidden layers for the T1-weighted MRI and TOF MRA features, respectively (refer to model architecture in Figure 2). For comparison purposes, a null model, which assigns the average age of the training datasets to all test datasets, was also evaluated.

For a more detailed feature importance analysis, the SmoothGrad saliency method (50) was applied to individual models for randomly selected participants, as described in Mouches et al. (29). This gradient-based saliency method allows us to evaluate importance directly in the input data space (i.e., with saliency maps in the case of CNN models and with feature importance in the case of MLP models).

### Model combination

To assess the benefits of combining both imaging modalities (T1-weighted MRI and TOF MRA) and both input data types (images or morphological hand-crafted features), different combinations of the four individually-trained models were evaluated: CNN based on T1-weighted data (CNN_T1), CNN based on TOF MRA data (CNN_TOF), MLP based on T1-weighted morphological hand-crafted features (MLP_T1), and MLP based on TOF MRA morphological hand-crafted features (MLP_TOF). Therefore, the predicted brain age results for the validation data for each individual model were used as input to a multiple linear regression model trained to predict brain age (refer to Figure 2), as proposed by Jonsson et al. (51). These weights were then also applied when linearly combining the test data from the 5-folds described above. Wilcoxon-signed ranked tests were used to compare the absolute errors on the test data of (i) the CNN_T1 vs. the CNN_T1+MLP_T1; (ii) the CNN_TOF vs. the CNN_TOF+MLP_TOF; and (iii) the CNN_T1+CNN_TOF vs. the model combining all four individual models. As a result, the benefits of adding morphological hand-crafted features for every single modality and in a multimodal setting are assessed.

### Brain age gap and association with cardiovascular risk factors

It is well-known that brain age prediction models suffer from age-related biases. These biases result in the age of young subjects typically being overpredicted and the age of elderly subjects typically being underpredicted (52, 53). Therefore, the predicted brain ages from each individual model (MLP_T1, MLP_TOF, CNN_T1, CNN_TOF) were first adjusted for age bias, following the linear regression method proposed in de Lange et al. (25). For each fold of the cross-validation, the validation data were first used to compute the slope (α) and intercept (β) of the regression line between chronological age and predicted age such that:


(1) $$\begin{array}{c} Predicted\;Age=\alpha \times Chronological\;Age+\beta \end{array}$$

After that, the predicted results of the test data were corrected as follows:


(2) $$\begin{array}{l} Corrected\;Predicted\;Age=Predicted\;Age+[Chronological\;Age\\ \;-\left(Chronological\;Age\times \alpha +\beta \right)] \end{array}$$

Finally, the BAG, representing the difference between the corrected predicted brain age and the chronological age, was computed for each test subject and individual model.

After age-related bias correction, associations between the BAG and cardiovascular risk factors were analyzed for the test subjects (not involved in model training or age-related bias parameters estimation). Multiple linear regression analyses were conducted for each cardiovascular risk factor and for each individual model BAG (MLP_T1, CNN_T1, MLP_TOF, CNN_TOF):


(3) $$\begin{array}{l} BAG=\beta_{1}\times Chronological\;Age+\beta_{2}\times Sex\\ \;+\beta_{3}\times Cardiovascular\;Risk\;Factor \end{array}$$

Here, the specific risk factors along with age and sex were used as independent variables while the brain age gap was used as the dependent variable (25). The *p*-values of the beta coefficients were corrected for false discovery rate (54) and *p*-values < 0.05 were considered significant. The cardiovascular risk factors were standardized prior to the multiple linear regression analyses to enable a comparison of the beta coefficients. The analyses were conducted using the python statsmodels package (55).

## Results

### Individual model performance

The performance of each individual model is reported in Table 2. It shows the mean absolute error (MAE) and Pearson's correlation (r) when comparing the chronological age and the estimated biological brain age, averaged over the five cross-validation folds. When analyzing each imaging modality separately, the CNN models perform better than the corresponding MLP models. Overall, more accurate results are seen for the models based on T1-weighted images, with the CNN using T1-weighted MRI images as input performing the best (MAE: 4.20 years). The worst performing model is the MLP using morphological hand-crafted features derived from the TOF MRA datasets (MAE: 9.52), although it is worth noting that it still outperforms the null model (MAE: 11.41).

**Table 2:** Performance of the null model and of the different individual and combined models.

	**Model**	**MAE (std across CV folds)**	**R (std across CV folds)**	**Age-related bias (r) (std across CV folds)**
Null model		11.41 (0.10)	−0.01 (0.0)	1 (0.0)
Individual models	MLP_T1	5.54 (0.20)	0.861 (0.012)	−0.432 (0.043)
	MLP _TOF	9.52 (0.26)	0.524 (0.025)	−0.790 (0.012)
	CNN_T1	4.20 (0.18)	0.926 (0.004)	−0.453 (0.059)
	CNN_TOF	5.13 (0.19)	0.887 (0.012)	−0.466 (0.080)
Combined models	Combined T1	4.11* (0.08)	0.929 (0.004)	−0.489 (0.046)
	Combined TOF	5.06* (0.22)	0.887 (0.012)	−0.476 (0.058)
	Combined MLP	5.49 (0.22)	0.865 (0.012)	−0.510 (0.026)
	Combined CNN	4.08 (0.13)	0.931 (0.005)	−0.491 (0.047)
	All combined	4.00^†^ (0.10)	0.932 (0.005)	−0.420 (0.040)

MAE, Mean absolute error (years); r, Pearson's correlation between the predicted and chronological age, Age-related bias: Pearson's correlation between the chronological age and the brain age gap. For each column, the average and standard deviation over the five cross-validation folds are reported. All Pearson's correlation values are significant (< 0.001, two-tailed *p*-value).

* Significant difference with the corresponding CNN (Wilcoxon signed ranked test; *p* < 0.01); ^†^Significant difference with the Combined CNN (Wilcoxon signed ranked test; *p* < 0.05). std, Standard deviation; CV, Cross-validation; MLP, Multilayer perceptron; CNN, Convolutional neural network; T1, T1-weighted MRI; TOF MRA, Time-of-flight magnetic resonance angiography.

**Figure 3:**
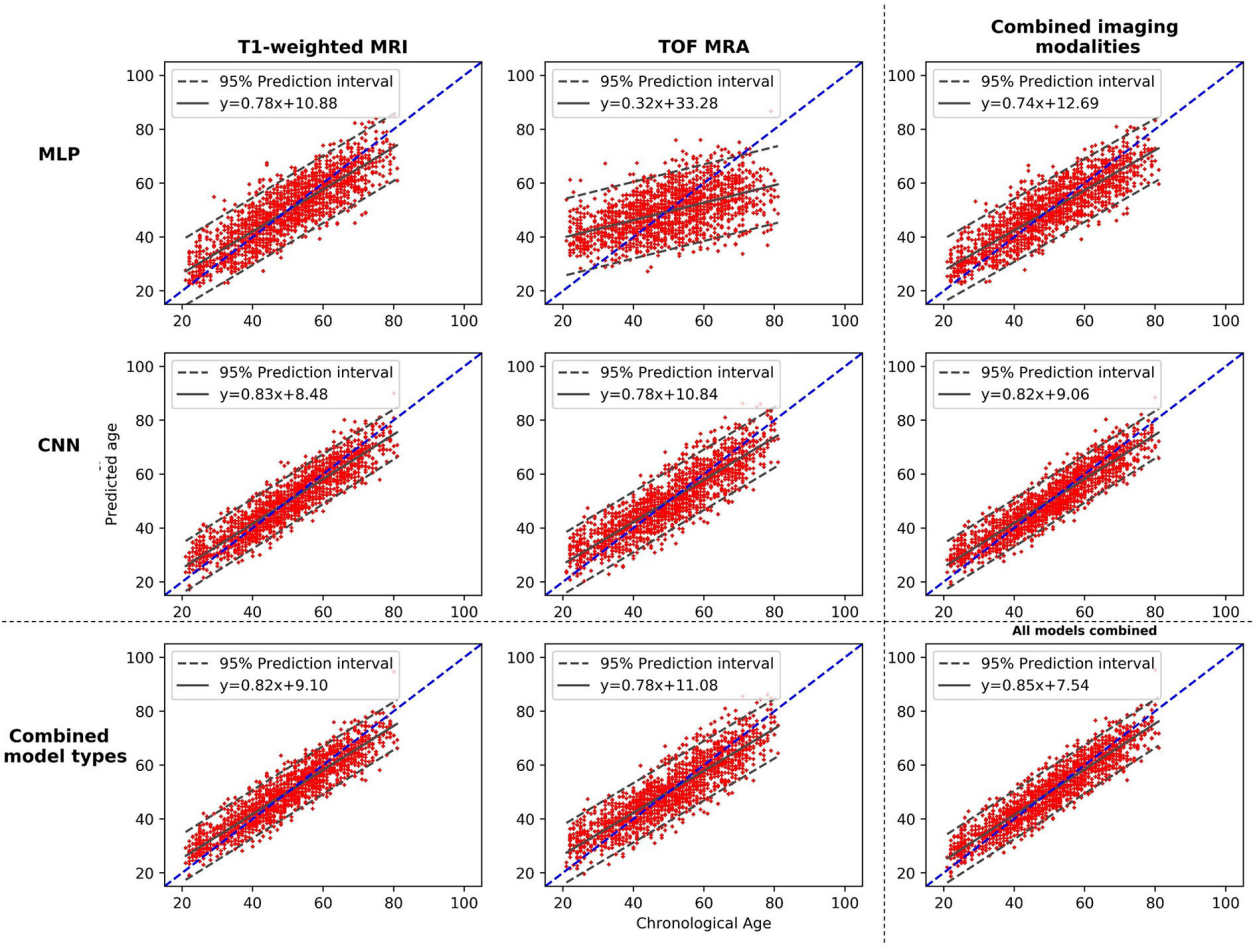
Plots of the brain age predictions vs. the chronological age for the different models and the different possible model combinations. The dashed line shows the diagonal, and the plain line shows the fitted regression line, with its equation. MLP, Multilayer perceptron; CNN, Convolutional neural network; T1-weighted MRI, T1-weighted magnetic resonance imaging; TOF MRA, Time-of-flight magnetic resonance angiography.

Figure 3 displays plots of the chronological age vs. the predicted age. It shows the performances of each individual model, of pairwise model combinations, and when all models are combined. More accurate models show a tighter 95% prediction interval around the fitted linear regression line. The fitted linear regression lines also clearly illustrate the age-related bias present in the model's predictions, which is further confirmed by the non-zero intercept value of each regression line. Overall, among the four individual models, the CNN_T1 model demonstrates the tightest 95% prediction interval and the MLP_TOF model the largest one.

**Figure 4:**
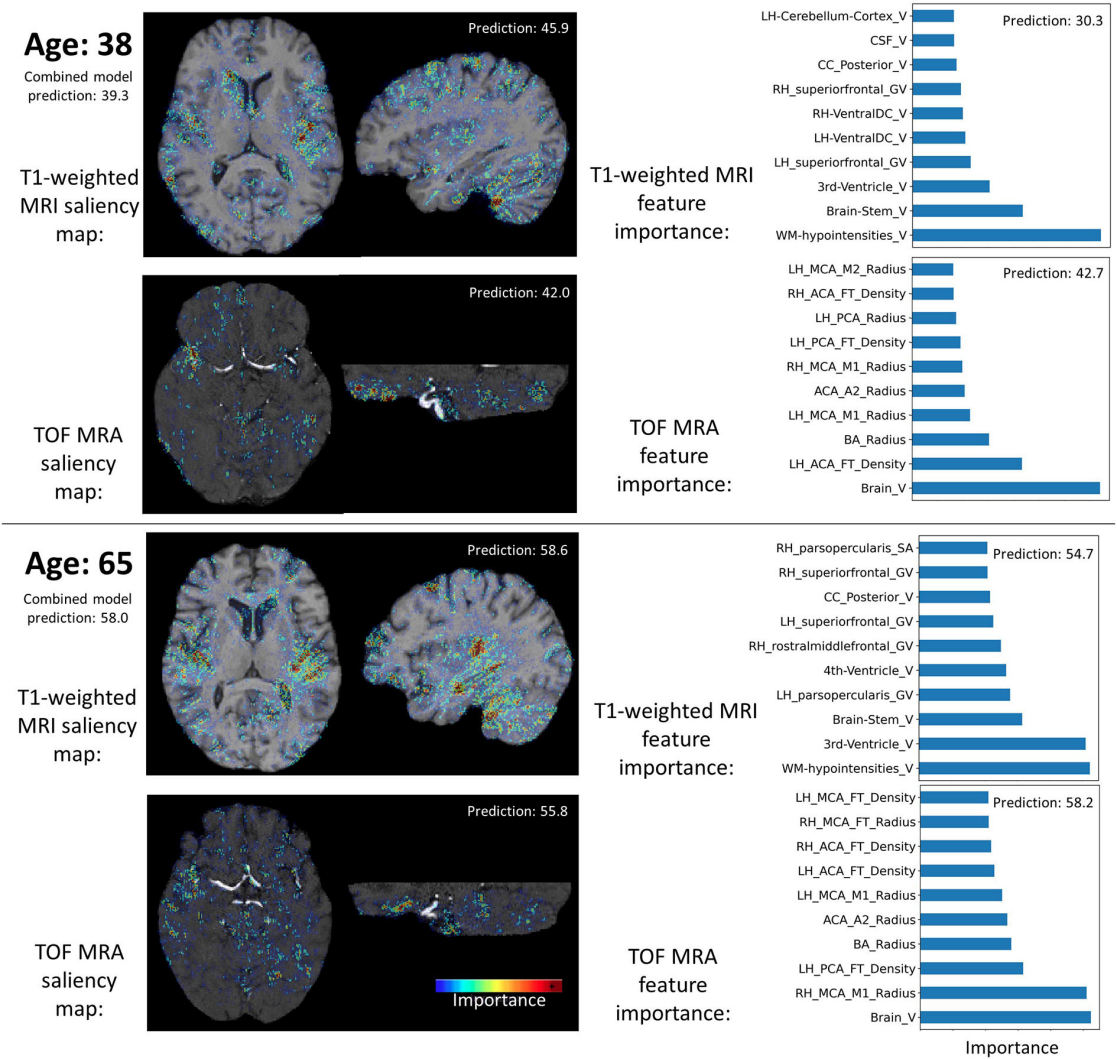
Examples of input data importance (axial and sagittal views of saliency maps for the CNN models and feature importance for the MLP models) for two participants. Brain age predictions of the combined and individual models are indicated. RH, Right hemisphere; LH, Left hemisphere; PCA, Posterior cerebral arteries; MCA, Middle cerebral artery; BA, Basilar artery; ICA, Internal carotid artery; FT, Flow territory; V, Volume; GV, Gray volume; TA, Thickness average.

Figure 4 shows saliency maps and feature importance for two test participants. These two examples demonstrate that the two data types (i.e., image and hand-crafted feature) bring complementary information. For instance, for the T1-weighted MRI data, the lateral sulcus are identified as important, in line with our previous study (29), while for the hand-crafted features, the white matter hypointensities, third ventricle, and brainstem appear as more important.

In summary, the individual model performances reveal that the CNN using T1-weighted images performs best. Moreover, it shows that all models and types of input data contain age-relevant information, as they all lead to better results compared to the null model.

### Combined model performance

When combining the two types of input data (MLP + CNN models), significant improvements are observed for both imaging modalities (CNN_TOF alone: MAE = 5.13 and CNN_TOF+MLP_TOF: MAE = 5.06, *p* < 0.01; CNN_T1 alone MAE = 4.20 and CNN_T1+MLP_T1: MAE = 4.11, *p* < 0.01). When evaluating cross-modality combinations, the model combining the output predictions of all four different models resulted in the best overall MAE of 4.0 years. This is a slight improvement compared with the results obtained when only the two CNNs were combined (MAE = 4.08; *p* < 0.05). The model combining all four individual models also resulted in the smallest age-related bias (*r* = −0.420), which is illustrated by the chronological age vs. predicted age plot shown in Figure 3. These results suggest that diversifying the type of input data has the potential to improve brain age prediction performances. They also illustrate that CNN models specifically designed for the brain age prediction task still benefit from being combined with hand-crafted feature-based models.

In terms of input data type importance, the weights of the linear combination can be used to get a general understanding of the importance of each modality. The normalized weights for each model averaged over the 5-folds, are CNN_T1: 0.62; CNN_TOF: 0.23; MLP_T1: 0.23; MLP_TOF: −0.08. These results are in line with individual model performances as best performing individual models also show the highest weights in the linear combination.

### Association with cardiovascular risk factors

**Figure 5:**
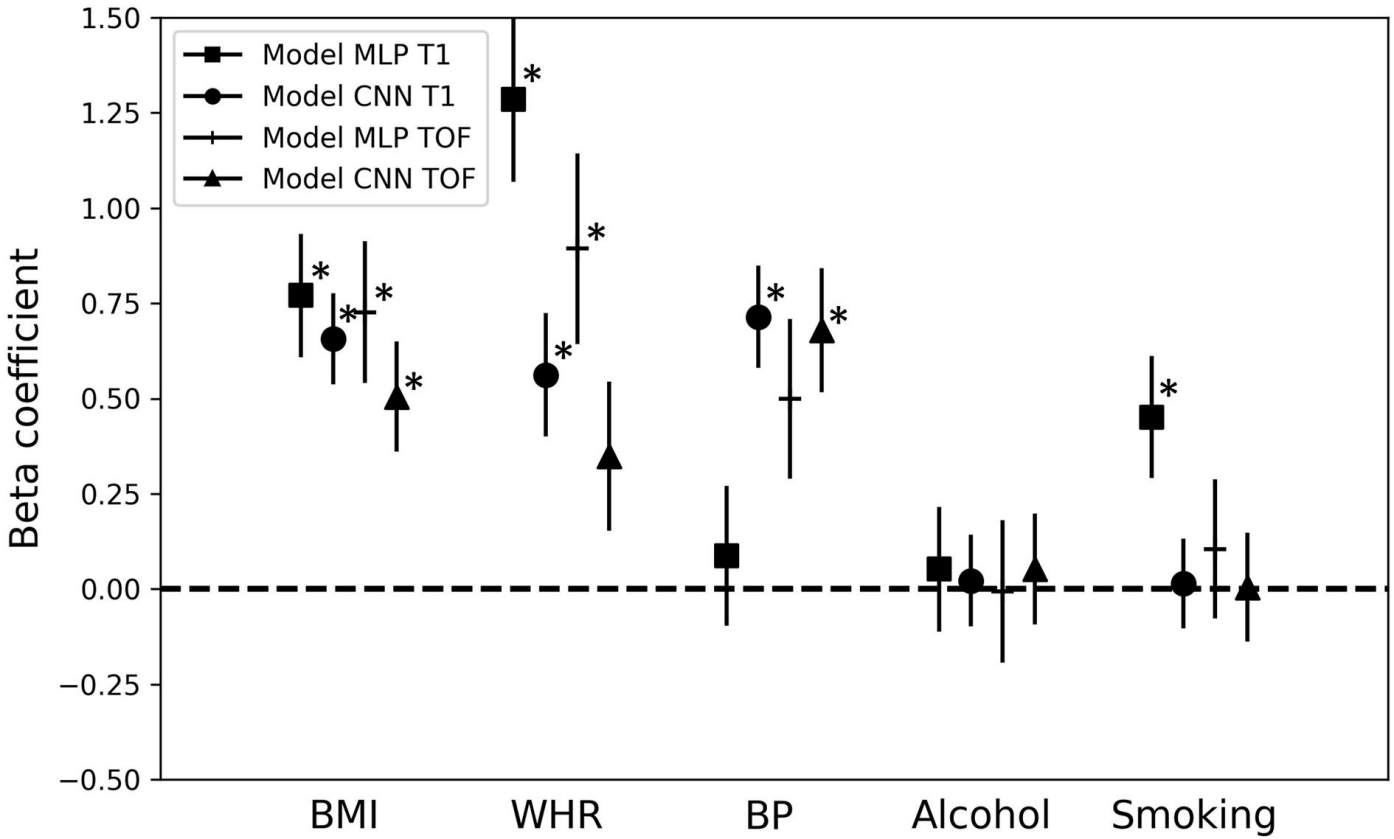
Association of the brain age gap with cardiovascular risk factors. Beta coefficients and their error bars for the different cardiovascular risk factors for each multiple regression model predicting the brain age gap for the different brain age prediction models. *p < 0.05; MLP, Multilayer perceptron; CNN, Convolutional neural network; T1, T1-weighted MRI; TOF, Time-of-flight MRA; BMI, Body-mass-index; WHR, Waist-to-hip ratio; BP, Blood pressure.

Figure 5 shows the beta coefficients for the different cardiovascular risk factors [i.e., β_3_ in Equation (3), Section Brain age gap and association with cardiovascular risk factors] and their standard errors as estimated by the multiple regression models. Significant beta coefficients (*p* < 0.05, after false discovery rate correction) are indicated with ^*^. BMI, WHR, and BP showed the highest correlations with the BAG. BMI was significantly associated with each of the four model BAGs (all β > 0.50). The two factors related to body fat (BMI and WHR) showed an overall greater association with the BAGs from the MLP models than with the BAGs from the CNN models (BMI: MLP BAGs β > 0.72, CNN BAGs β < 0.66; WHR: MLP BAGs β > 0.88, CNN BAGs β < 0.57). BP was significantly associated with the BAG from the CNN models (CNN BAGs β > 0.67), while smoking showed a significant correlation only for the MLP_T1 BAG (β = 0.45). Alcohol consumption did not show any significant association with any of the BAGs. BMI and WHR showed significant association with the BAG from the MLP models, but lower beta coefficients for the BAG from the CNN models. On the other hand, BP had higher beta coefficients for the BAG from the CNN models. These discrepancies observed for the different types of models may be explained by the type of information contained in the input data.

## Discussion

This study demonstrates that combining outputs from brain age predictive models using diverse types of input data (morphological hand-crafted features and images) leads to significantly increased prediction accuracy. More specifically, these findings hold true for (i) two different imaging modalities, and (ii) in a multimodal context. Furthermore, associations between the BAGs obtained using the different imaging modalities and input data types and several cardiovascular risk factors were identified. This illustrates that BAGs computed using different brain age prediction models capture different information regarding cardiovascular risk factors (refer to Section Association with cardiovascular risk factors).

### Brain age-prediction model performance

Overall, the results from this study show that self-optimized CNN models achieve more accurate brain age predictions compared to MLP models using morphological hand-crafted features. This behavior was observed for both imaging modalities and is in line with previous studies using T1-weighted MRI data and derived features (19, 20). Additionally, this study demonstrates that the same behavior can be found when using a completely different imaging technique, namely, TOF MRA datasets. It is likely that this finding is also true for other sequences. The second finding of this study shows that models using the brain tissue information from T1-weighted MRI data perform better than the two models using brain artery information from TOF MRA data. This was also reported in our previous study using CNN models (29). The MLP_TOF model shows the worst results but performs better than the null model. This confirms that measurements of brain arteries contain at least some relevant age-related information, as previously observed in Mouches et al. (6). This low performance could also be explained by the small number of input features (25 artery measurements) fed to the MLP_TOF. The considerable difference between the results of the CNN_TOF and MLP_TOF models suggests that the CNN_TOF model uses additional information, other than artery density and thickness. This information is potentially not directly related to the arteries, although brain tissue contrast is poor on TOF MRA data. In line with this finding, it was reported in our previous study (29) that cerebrospinal fluid structures, visible on TOF MRA datasets, were also used by the CNN models for decision making. Therefore, it may be argued that, first, the CNN_TOF model also used some tissue morphology information from the TOF MRA datasets and, second, that using only artery-related information for brain age prediction as done by the MLP_TOF model does not enable an accurate age prediction.

The accuracy of the biological brain age prediction improves when combining the predictions from the CNN models, automatically identifying optimized features, and the MLP models, using morphological hand-crafted features. These results are generally in line with the findings of Bermudez et al. (19) and (20). The former aggregated brain volumetric features and features extracted by a CNN model using T1-weighted MRI data. The latter ensembled the output predictions from seven different models using diverse machine learning algorithms/deep learning model architectures and using various input data types (hand-crafted features, gray and white matter maps) derived from T1-weighted MRI datasets. Both studies reported higher accuracy when aggregating both feature types compared to using only the volumetric features or only the CNN features. Compared to those previous studies, the current study adds relevant new insights for biological brain age prediction methods. First, the CNN architecture used in this study is one of the best performing, state-of-the-art architectures for brain age prediction (13). Therefore, our findings demonstrate that even such advanced CNN models designed for the specific task of brain age prediction can benefit from being coupled with models using morphometric features. Second, two imaging modalities providing complementary information were considered in this study, extending these findings beyond just T1-weighted MRI datasets. Similarly, past studies showed that ensembling predictions from CNN models trained on different inputs derived from T1-weighted MRI scans (e.g., gray matter and white matter segmented images, linearly and non-linearly registered images, etc.) lead to improved brain age prediction accuracy (13, 23, 51). Overall, all of these findings support the notion that combining different data representations from a single imaging modality has advantages, even when using deep learning models. Indeed, CNN-based features are abstract, high-level features, and do not necessarily match clinically relevant features. Similarly, some morphological features such as cortical thickness cannot be easily replaced by imaging features that are purely derived using convolutions.

For instance, in Levakov et al. (56), a brain age prediction CNN model based on T1-weighted MRI scans was implemented and brain regions contributing the most to the model's predictions were identified. The authors reported that the fourth ventricle, among other regions, was a predictive region used by CNN. However, they also found that the volume of the fourth ventricle was not correlated with age (56). In their study, some structures where morphology is known to be correlated with age were not used by their CNN. This is the case for the frontal pole, for instance, whose thickness is known to be highly affected by age (2, 26, 57). These observations and the saliency analyses suggest that combining clinical knowledge from derived morphological features such as cortical thickness and knowledge from trained machine learning models can potentially lead to improved biological brain age estimation. Feature importance could also be investigated in the context of an end-to-end trained combined model to investigate the relationships between the different input data types. Nevertheless, implementing explainability methods in a combined model using different data types and model architectures is not straight forward and needs further research itself (58).

### Association with cardiovascular risk factors

Relevant associations between the risk factors and the BAGs were found. More precisely, increased BMI, WHR, and BP were associated with an increased BAG (i.e., predicted age > chronological age; older-looking brain). These results are in line with previous research reporting a negative impact of these factors on brain tissue atrophy, white matter lesions (59), and artery density and thickness (6, 60). Moreover, similar associations were also identified in the past when predicting brain age from morphological hand-crafted features. Past studies also reported associations with BP and smoking status (24, 25, 33, 34), as well as with obesity markers (33, 34, 61).

The differences observed in the associations between cardiovascular risk factors and the CNN vs. the MLP BAGs could be explained by the differences captured in the input data. For example, the CNN models used preprocessed images that were affinely registered to a template, which removes some information from the images such as the total brain volume. However, this information might be better captured in the morphological hand-crafted features containing raw volumes of brain structures. Other important information such as white matter lesions, appearing hypointense on T1-weighted MRI data, might be better represented in the image data. Although these lesions are segmented by FastSurfer and their volume is used as a hand-crafted feature, the information about their location is not encoded in the hand-crafted features. Therefore, BAGs obtained from brain age estimation based on different data types (hand-crafted features *vs*. images) or imaging modalities contain different information and biases. It is also important to note that image preprocessing, such as the type of image registration applied, can influence the information contained in the image. Thus, it can affect the model performance as well as the association of the BAG with other factors. For these reasons, it would be interesting to investigate the influence of pre-processing steps on brain age prediction accuracy in more detail. Klingenberg et al. (60), for example, investigated the influence of linear and non-linear image registration used for preprocessing of datasets on the accuracy of neurological disease classification. They found that the registration of datasets to a common reference space as a pre-processing step generally improves classification accuracy. It is likely that this is also the case for the brain age prediction task as the machine learning models need to learn other correction factors in case of non-registered datasets (e.g., orientation and location of the brain) that are not related to the brain age but increase the complexity of the training task.

### Limitations

This study has a few limitations that should be mentioned. First, this study used cross-sectional data and cardiovascular risk factors that were collected at a single time point in life. However, the exposure duration to risk factors might considerably impact the BAG. Thus, including temporal data could also reveal if changes in the brain caused by exposure to risk factors are reversible. Moreover, this study was limited to an analysis of selected cardiovascular risk factors. However, the BAG biomarker has been shown to be associated with more diverse factors, such as medical history (35) and genetics (51), which should also be taken into account in future studies. Next, some limitations regarding the fairness of the models' comparison remain. Such limitations include the input data dimensionality, which differs between the T1-weighted MRI- and TOF MRA-based models, and the image preprocessing steps. Those were applied prior to the analyses with CNNs but not prior to the feature extraction process. Therefore, the impact of these two aspects on brain age prediction model performance should be studied in the future study. Finally, the different models were combined using a linear combination of their predictions, which led to improved accuracy. Nevertheless, more sophisticated, and potentially non-linear, methods should be investigated in the future as ways to further improve the results presented in this study. In terms of the association of the BAG with cardiovascular risk factors, linearly combining model outputs results in a weighted average of the cardiovascular risk factors-related information contained in the different data sources used to generate the BAGs. Therefore, using a non-linear combination could help to identify more complex interaction patterns between the information captured by each data source or to mitigate the biases captured in the BAGs.

## Conclusion

In conclusion, the results of this study demonstrate the benefits of using diverse sources of input data for the brain age prediction task. Doing so has the potential to improve the quality of the brain age gap biomarker and its ability to capture biologically relevant deviations from normal brain aging patterns. Moreover, associations between the brain age gap and factors impacting normal brain aging trends should be carefully interpreted. The current findings demonstrate that these associations rely on the information captured by the data, and on the data preprocessing methods. Therefore, bringing together clinical knowledge and advanced black box deep learning could help toward extracting more diverse data and, thus, generate better brain aging models.

## Data availability statement

The data analyzed in this study was obtained from the Study of Health in Pomerania (SHIP; https://www2.medizin.uni-greifswald.de/cm/fv/ship/), the following licenses/restrictions apply: The options for using data from SHIP are described in the “Regulations on the use and handling of data and sample material”. The statistical evaluations should be carried out taking into account the “Guidelines for quality-assured evaluation”. Requests to access these datasets should be directed to SHIP, https://www2.medizin.uni-greifswald.de/cm/fv/ship/daten-beantragen/.

## Ethics statement

The studies involving human participants were reviewed and approved by the local ethics commission of the University of Greifswald (BB 39/08, 19.06.2008). The patients/participants provided their written informed consent to participate in this study.

## Author contributions

PM, MW, and NF designed the study and interpreted the results. PM performed the research, data analysis, and drafted the manuscript. AA performed data analysis. SL collected the data. All authors revised the manuscript and approved the submitted version.
